# Evaluation of Multiple RNA Extraction Protocols for Chikungunya Virus Screening in *Aedes aegypti* Mosquitoes

**DOI:** 10.3390/ijms25126700

**Published:** 2024-06-18

**Authors:** Bárbara Caroline Garcia Freitas, Daniel Damous Dias, Lúcia Aline Moura Reis, Leonardo Henrique Almeida Hernández, Glennda Juscely Galvão Pereira Cereja, Carine Fortes Aragão, Sandro Patroca da Silva, Joaquim Pinto Nunes Neto, Carmeci Natalina Elias, Ana Cecília Ribeiro Cruz

**Affiliations:** 1 Parasite Biology in the Amazon Region Graduate Program, Pará State University, Belém 66087-670, PA, Brazil; barbaracarolinefreitas@hotmail.com (B.C.G.F.); damous1994@gmail.com (D.D.D.); luciaalinereis@gmail.com (L.A.M.R.); joaquimneto@iec.gov.br (J.P.N.N.); 2 Department of Arbovirology and Hemorrhagic Fevers, Evandro Chagas Institute, Health and Environment Surveillance Secretariat, Ministry of Health, Ananindeua 67030-000, PA, Brazil; leohenrique96@hotmail.com (L.H.A.H.); glenndajgp@gmail.com (G.J.G.P.C.); carinefaragao@gmail.com (C.F.A.); spatroca@gmail.com (S.P.d.S.); 3 Goiás Public Health Laboratory, Goiânia 74853-120, GO, Brazil; carmecielias@gmail.com

**Keywords:** *Aedes*, chikungunya virus, molecular testing, RNA extraction

## Abstract

*Chikungunya virus* (*Togaviridae*, *Alphavirus*; CHIKV) is a mosquito-borne global health threat. The main urban vector of CHIKV is the *Aedes aegypti* mosquito, which is found throughout Brazil. Therefore, it is important to carry out laboratory tests to assist in the virus’s diagnosis and surveillance. Most molecular biology methodologies use nucleic acid extraction as the first step and require quality RNA for their execution. In this context, four RNA extraction protocols were evaluated in *Ae. aegypti* experimentally infected with CHIKV. Six pools were tested in triplicates (n = 18), each containing 1, 5, 10, 20, 30, or 40 mosquitoes per pool (72 tests). Four commercial kits were compared: QIAamp^®^, Maxwell^®^, PureLink^®^, and PureLink^®^ with TRIzol^®^. The QIAamp^®^ and PureLink^®^ with TRIzol^®^ kits had greater sensitivity. Two negative correlations were observed: as the number of mosquitoes per pool increases, the Ct value decreases, with a higher viral load. Significant differences were found when comparing the purity and concentration of RNA. The QIAamp^®^ protocol performed better when it came to lower Ct values and higher RNA purity and concentration. These results may provide help in CHIKV entomovirological surveillance planning.

## 1. Introduction

Infectious diseases caused by arthropods are known as arboviruses. Arboviruses replicate in arthropods’ tissues and are transmitted during blood feeding to vertebrates [1]. Most arboviruses of public health concern belong to the *Orthoflavivirus*, *Alphavirus*, or *Orthobunyavirus* genera and are often associated with major epidemics such as *Orthoflavivirus flavi* (YFV), *Orthoflavivirus zikaense* (ZIKV), *Orthoflavivirus denguei* (DENV), and *Chikungunya virus* (CHIKV), all transmitted by *Aedes aegypti*, which is the main vector in urban areas in Brazil [2].

Classified in the *Togaviridae* family, *Alphavirus* genus, CHIKV is an arbovirus that has an enveloped icosahedral nucleocapsid of 60 to 70 nm in diameter and a positive-sense single-stranded RNA with a genome of 11.8 Kb [3]. CHIKV was first identified in Tanzania in 1952 after the virus’s isolation from a patient’s serum. Within a few weeks, more than half of the population in this region developed symptoms of fever, rash, and arthralgia. Because of the stooped posture and stiff gait of infected individuals, the disease was named chikungunya, a word from the Kimakonde language which means “that which bends up” [4,5].

*Aedes* (*Stegomyia*) *aegypti* Linnaeus 1762 belongs to the order Diptera, infra-order Culicomorpha, family Culicidae, and genus *Aedes*. For a long time, *Ae*. *aegypti* was confined to the African continent [6]. Globalization and environmental changes, including climate change, are the main factors driving the geographical expansion of this species to other continents [7,8]. For tracking down hosts, female mosquitoes have several physiological, morphological, and chemical mechanisms that identify signals such as smell, heat, and CO_2_. *Ae*. *aegypti* have a high degree of synanthropy and can fully develop their life cycle in urban and domestic environments. This species is also diurnal, which intensifies the human–mosquito relationship in the urban environment [9,10].

Most molecular biology methodologies use nucleic acid extraction as the first step. It is possible to obtain RNA from sundry biological sources, and there are multiple protocols and reagents available to carry out this procedure for different purposes [11]. Furthermore, the applied protocol must be efficient in generating good-quality and concentrated RNA without amplification inhibitors or contaminants such as proteins, carbohydrates, and other nucleic acids [11,12].

The reverse transcription (RT) assay followed by real-time polymerase chain reaction (qPCR) is one of the most widely used detection methods due to its sensitivity and specificity through the use hydrolysis probes. Some studies have demonstrated the efficiency of the molecular diagnostic method through RT-qPCR based on entomological samples. It has also become a very useful tool for epidemiological and entomovirological surveillance [13,14].

There is minimal information available on the use of these RNA extraction commercial kits for insect samples, as well as a lack of studies demonstrating the efficiency and quality of extraction in this kind of sample. Also, considering that once infected, mosquitoes remain infected for the rest of their lives and can transmit the virus to several hosts, here, we evaluated the effectiveness and quality of four different RNA extraction protocols in *Ae*. *aegypti* experimentally infected with CHIKV.

## 2. Results

### 2.1. RNA Extraction Protocols Performance Comparison by RT-qPCR

The performance of CHIKV detection was evaluated in six different pools containing 1, 5, 10, 20, 30, or 40 mosquitoes extracted via four different protocols. Each pool was tested in triplicates (A, B, and C), totaling 72 tests. The RT-qPCR results showed that pools extracted via QIAamp^®^ and the PureLink^®^ associated with TRIzol^®^ methods had CHIKV RNA detected in all 18 reactions. On the other hand, the RNAs extracted via Maxwell^®^ and regular PureLink^®^ kits were positive for CHIKV in 8 and 13 reactions, respectively (Table 1 and Table S1).

As described, the Maxwell^®^ and PureLink^®^ data groups have undetected results. To normalize the data, we compared only the groups with totally detected samples (QIAamp^®^ and PureLink^®^ with TRIzol^®^). The parameters of normality, variances’ homogeneity, and outliers’ absence were checked to choose the appropriate test for comparing two independent samples.

**Figure 1:**
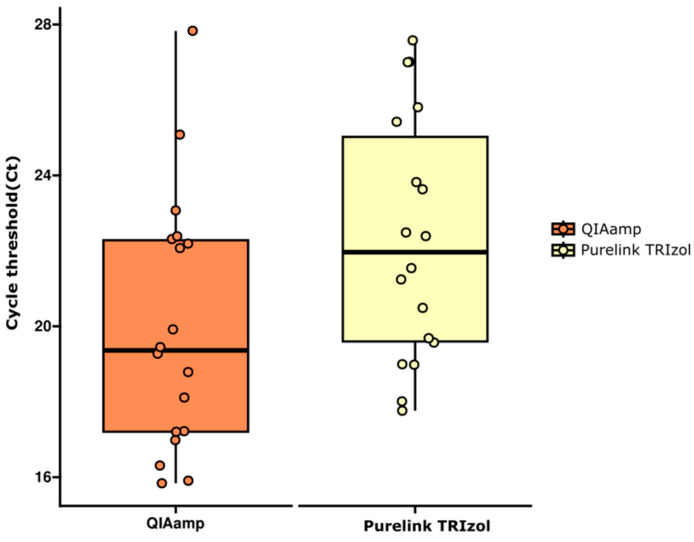
Comparison of CHIKV detection in RNA extracted samples from *Ae. aegypti* pools via QIAamp^®^ and PureLink^®^ with TRIzol^®^ methods.

Since the data had a normal distribution, variance homogeneity, and no outliers, a parametric test to compare means for independent samples (Student’s *t*-test) was used to see if there was a difference between the means of both data groups. The test indicates a significant difference between them (t = −2.0853; *p* = 0.04462) (Figure 1).

Given that the two protocols did not have a normal distribution, Spearman’s non-parametric correlation test was applied to assess the relationships among the analyzed variables. The correlation test results for the number of mosquitoes and the Ct value is shown at Table 2.

Two negative and statistically significant correlations were observed: one between number of mosquitoes per pool and QIAamp^®^ (ρ = 0.749; *p*-value = 0.0003); and the other between number of mosquitoes per pool and PureLink^®^ with TRIzol^®^ (ρ = −0.542; *p*-value = 0.020). These results indicate that as the number of mosquitoes per pool increases, the Ct value decreases.

### 2.2. RNA Purity Comparison

The ratio of 260/280 nm was used to analyze the RNA purity degree in the samples. Analyzing the RNA purity obtained via the different extraction methods, the one that resulted in the highest degree of RNA purity, i.e., a greater number of samples with values greater than or equal to 1.8 in the 260/280 nm ratio, was the QIAamp^®^ kit. Due to the lack of normality in most of the variables, the non-parametric Kruskal–Wallis test was used to compare the values among the groups, followed by Dunn’s post hoc test with Bonferroni correction for multiple comparisons.

**Figure 2:**
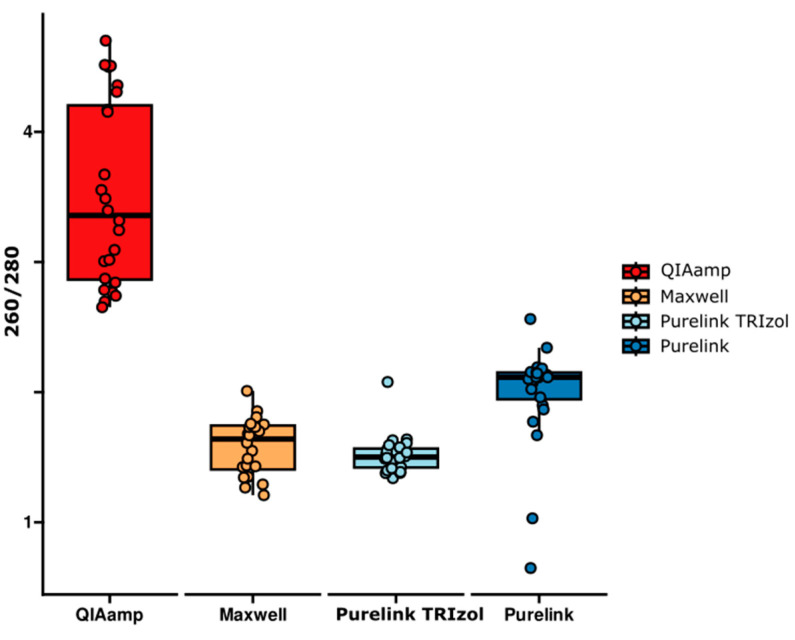
RNA purity degree (260/280 nm) comparison by RNA extraction protocol.

The Kruskal–Wallis test indicated significant differences among the groups (χ^2^(2) = 70.6325; *p* = 3.124733 × 10^−15^) (Figure 2). Dunn’s post hoc analysis with Bonferroni correction revealed that the QIAamp^®^ method showed statistically significant differences in relation to the Maxwell^®^ (*p* = 5.507 × 10^−11^), PureLink^®^ with TRIzol^®^ (*p* = 4.390 × 10^−13^), and PureLink^®^ (*p* = 1.863 × 10^−3^) methods. On the other hand, the Maxwell^®^ method showed a significant difference compared to the PureLink^®^ protocol (*p* = 7.895 × 10^−3^). Finally, the PureLink^®^ with TRIzol^®^ kit showed a significant difference in relation to the PureLink^®^ method (*p* = 6.378 × 10^−4^).

### 2.3. RNA Quantification Comparison

Both RNA quantification methods comparison were assessed via the same statistical tests applied to the purity analysis. Kruskal–Wallis test was used to compare the means among groups, and Dunn’s post hoc test with Bonferroni correction was used to identify significant differences among specific pairs of groups.

**Figure 3:**
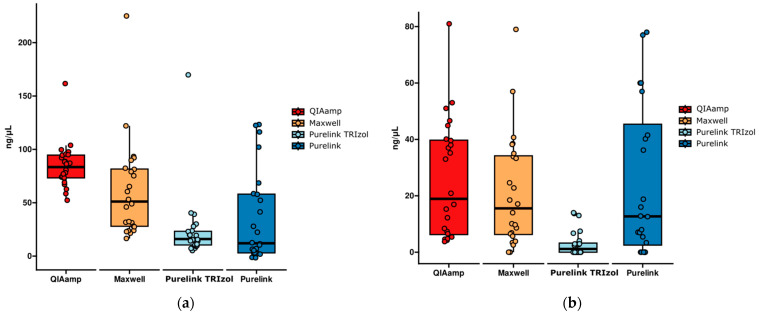
Comparison of RNA concentration (ng/µL) assessed via (**a**) NanoDrop^®^ and (**b**) Qubit equipment.

In the analysis of the NanoDrop^®^ RNA concentration results, the Kruskal–Wallis test indicated significant differences among the four kits (χ^2^(2) = 39.0748; *p* = 1.673494 × 10^−8^) (Figure 3a). Dunn’s post hoc analysis with Bonferroni correction showed that the QIAamp^®^ method presented statistically significant differences in relation to the PureLink^®^ with TRIzol^®^ (*p* = 2.243 × 10^−7^) and PureLink^®^ (*p* = 8.227 × 10^−6^) protocols. On the other hand, the Maxwell^®^ kit showed a significant difference in relation to the PureLink^®^ with TRIzol^®^ (*p* = 2.986 × 10^−3^) and PureLink^®^ (*p* = 2.988 × 10^−2^) methods.

Likewise, the Kruskal–Wallis test indicated significant differences among the groups (χ^2^(2) = 27.0534; *p* = 5.737462 × 10^−6^) in the Qubit results comparison (Figure 3b). Dunn’s post hoc analysis with Bonferroni correction showed that the QIAamp^®^ method presented a statistically significant difference in relation to the PureLink^®^ with TRIzol^®^ protocol (*p* = 1.355 × 10^−5^). On the other hand, the PureLink^®^ with TRIzol^®^ method showed a significant difference in relation to the Maxwell^®^ (*p* = 3.717 × 10^−4^) and PureLink^®^ (*p* = 9.856 × 10^−4^) kits. There were no statistically significant differences between the other methods.

## 3. Discussion

To be reliable and meaningful, any legitimate RNA extraction protocol must achieve specific goals. There are several different ways of extracting RNA from different biological sources, with the aim of producing a representative, high-quality sample [15]. Although RNA extraction methods for molecular detection are widely used in entomovirological surveillance, there are scarce available data concerning these kits for entomological samples that demonstrate the efficiency and quality of extraction.

In our study, when the Ct values were evaluated, the QIAamp^®^ protocol obtained lower values than the other methods, which may indicate greater sensitivity in detecting the target, generally related to the purity and concentration of RNA. The *t*-test indicated a significant difference between QIAamp^®^ and PureLink^®^ with TRIzol^®^ kits (*p* = 0.04462). The QIAamp^®^ kit uses a silica membrane, and samples are lysed in a buffer containing RNase inhibitors, usually guanidine salts. Centrifugal force allows the lysate to apply to the membrane to bind the nucleic acids [16,17].

The study carried out by Ahmed et al. (2022) [18] used the QIAamp^®^ protocol on *Ae*. *aegypti* mosquitoes to test for the four DENV serotypes. All samples were positives according to RT-qPCR, and the tests were sensitive when used to detect individual infected mosquitoes and mixed groups with infected and non-infected mosquitoes. These results are like ours, as RNA extraction via the QIAamp^®^ kit demonstrate high efficiency. Other studies have also used the same method [19,20,21,22].

The Maxwell^®^ kit uses an automated system designed to extract total viral RNA from human plasma or serum samples based on a magnetic bead separation technique. Its disadvantage is the potential transport of magnetic particles in eluted samples [17,23]. However, so far, no formal evaluation of RNA extraction from mosquitoes’ samples using this system has been reported. Previous studies have shown that viral RNA recovery from various kinds of samples, i.e., automated systems based on magnetic particles, can be inferior or superior to manual column techniques. The manual method also contributed to better overall analytical precision, as evidenced by lower coefficients of variation [24,25].

The PureLink^®^ kit also uses a silica membrane and provides rapid column-based purification of RNA from a wide range of cell types and tissues. This kit, when combined with TRIzol^®^ reagent, maintains RNA integrity while disrupting cells and dissolving cellular components. In addition, the TRIzol^®^ reagent provides immediate and highly effective inhibition of RNase activity during sample homogenization [26]. In this study, diagnostic sensitivity increased with the use of silica membranes after the use of TRIzol^®^ when compared to the use of the regular PureLink^®^ protocol.

Moreover, the PureLink^®^ with TRIzol^®^ protocol requires a different sample incubation time to the regular method for the dissociation of nucleoprotein complexes. This time may have been necessary for adequate RNA lysis in mosquitoes’ samples. Other studies have reported that TRIzol^®^ on a silica column also increases the sensitivity of the detection of virus-infected cell lines and tissues, as well as causing enveloped virus inactivation [27,28].

About the number of *Ae*. *aegypti* mosquitoes, two kits had negative correlations (QIAamp^®^ and PureLink^®^ with TRIzol^®^); i.e., as the number of mosquitoes per pool increased, the Ct value decreased. The former had a strong correlation and the latter a moderate one. The Maxwell^®^ and PureLink^®^ kits had very weak and weak correlations, respectively. Despite showing negative correlations, these did not reach statistical significance, possibly due to the absence of positive values in these data groups, which could have considerably influenced the correlation coefficient and the statistical significance of the test. In this context, the authors explain that the amount of fluorescence released is correlated with the concentration of the target in the original sample and inversely proportional to the number of cycles required to reach the system’s specific threshold and be considered positive [29,30].

Knowing that samples extracted by all kits received the same thawing pattern on ice, and that two kits had positivity in all samples, it is possible to state that the pool preparation process in this research, up to the separation of the aliquots, did not create bias. RNA could be degraded due to various conditions from pool preparation to molecular detection, so the integrity of RNA seems to have been preserved [31,32].

In the RNA purity comparison, only the QIAamp^®^ protocol was within the expected values in all samples. To other kits, some samples had values below the expected, corroborating the statistical results. Many factors may have influenced this, such as contamination by components of the method’s own solutions, such as residual guanidine, which is normally present in kits based on binding columns, such as the one used. Well-known contaminants, such as ribonucleases (RNases), converge more strongly on larger pooled samples, thus widely digesting the RNA [33].

The literature generally indicates purity values (A260/A280 nm) between 1.8 and 2.2. Although the purity values of the QIAamp^®^ kit exceeded the reference values, some studies indicate that kits with RNA carriers tend to have higher purity values as they enable a higher concentration of genetic material to be recovered during the process [34,35,36]. A low A260/A280 nm ratio is probably due to phase mixing when removing the upper aqueous phase from the TRIzol^®^ separation, and is also more common in samples with a very low RNA yield. This may explain why the PureLink^®^ with TRIzol^®^ obtained significant differences in purity between almost all the protocols, even though Phasemaker^®^ tubes were used for RNA separation.

The RNA concentration results obtained using the NanoDrop^®^ equipment indicate statistically significant differences, with the QIAamp^®^ kit showing highly significant differences in relation to the PureLink^®^ with TRIzol^®^ and regular PureLink^®^ methods. The Maxwell^®^ method showed a significant difference in relation to the PureLink^®^ with TRIzol^®^ (very significant) and PureLink^®^ (significant) methods. Although the PureLink^®^ with TRIzol^®^ method resulted in low concentrations, it proved to be more sensitive than the Maxwell^®^- and PureLink^®^-extracted RNA’s. NanoDrop^®^ RNA concentration measurement ranges from 1.6 ng/μL to 22,000 ng/μL.

Overall, the QIAamp^®^ kit showed a higher amount of RNA by Qubit compared to the other protocols in most samples. Even though very small amounts of RNA can be amplified, RNA yield is an important pre-analytical factor that determines the success of molecular analysis. The Qubit RNA concentration measurement ranges from 250 pg/µL to 100 ng/µL. Qubit and NanoDrop^®^ may be used together to determine the concentration of RNA or DNA, considering that NanoDrop^®^ also shows the presence of contaminants.

According to information from the manufacturers, it was possible to estimate the cost of the methods in dollars per sample. In this sense, the QIAamp^®^ kit is the second most economical compared to the others tested, which is another advantage. It also proved efficient in detecting CHIKV in the pools, as did the PureLink^®^ with TRIzol^®^ method. However, the QIAamp^®^ kit showed better performance in the detection of lower Cts and better quality and concentration of the extracted RNA. The Maxwell^®^ and regular PureLink^®^ methods showed less-robust results compared to the others (Table 3).

## 4. Materials and Methods

### 4.1. Viral Titration

The isolate PER160/H803609, obtained from a CHIKV-positive human blood sample (Asiatic genotype), was provided by the Viral Isolation Laboratory of the Department of Arbovirology and Hemorrhagic Fevers (SAARB) of the Evandro Chagas Institute (IEC) for viral stock, which was prepared using Vero cells.

Vero cells were prepared in a 24-well plate with maintenance culture medium 199 (Gibco, Grand Island, NY, USA), supplemented with 2% fetal bovine serum (FBS) and antibiotics (100 IU/mL penicillin and 100 µg/mL streptomycin). The plate was incubated in a CO_2_ oven (5%) at 37 °C for 48 h before inoculation [37].

Viral titration by the stock was carried out with a viral suspension serially diluted from 10^−1^ to 10^−12^ [38]. Growth culture medium 199 (Gibco) of the 24-well plates was substituted and 100 µL of the diluted viral sample was inoculated in duplicate into the respective wells with the half-confluent monolayers. Plates were incubated in a CO_2_ oven (5%) for one hour at 37 °C, gently shaking every 15 min for adsorption. After, 3 mL of carboxymethylcellulose (CMC, 3% in medium 199) supplemented with 5% FBS, penicillin (100 IU/mL), and streptomycin (100 µg/mL) was added to each well, and plates were re-incubated in the CO_2_ oven (5%) for four days.

Then, cells were fixed by adding 3 mL of 10% formaldehyde and incubated at room temperature for four hours. After this time, plates were washed in running water, 3 mL of 0.1% crystal violet dye was added to each well, and they were incubated for 24 h at room temperature. Based on the number of plaque-forming units (PFU), considered to be positive for the characteristic cytopathic effect (CPE) in Vero cells, the viral titer was calculated using the method established by [38,39], where it is expressed in plaque-forming units per milliliter (PFU/mL).

### 4.2. Rearing and Maintenance of Ae. aegypti Colonies

The *Ae. aegypti* colonies were maintained and experimentally infected at the Medical Entomology Laboratory of the SAARB/IEC.

Eggs used for colony maintenance were from the F0 generation, while their prole (F1 generation) was used for the experimental infection. Eggs were placed in a basin with water and fish feed, as a source of food for the larvae, until hatching. The larval stage lasts six to eight days, after which they develop into pupae, a stage that lasts an average of two days until the mosquitoes’ emergence into their adult form.

In their adult stage, the mosquitos were transferred to entomological cages and fed bee honey diluted in 10% destilled water. The cages and mosquito basins were maintained in the insectary at 28 °C ± 1 °C with 80% ± 10% relative humidity, under a 12 h light/12 h dark cycle. Some female mosquitoes were used for the experimental infection and for colony maintenance.

The group in which the prole was infected were fed hamsters (*Mesocricetus auratus*) blood. They were previously anesthetized with tiletamine + zolazepam (Zoletil^®^ 50) at a dosage of 1500 mg/kg and placed in the vivarium for 15 min. The laid eggs were placed on filter paper and stored at room temperature in small acrylic boxes labeled with the date, the mosquito species, and the generation.

### 4.3. Experimental Infection

The oral infection procedure followed the protocol described by [40]. Five infections were carried out for the group with a viral titer of 1.45 × 10^7^ PFU/mL. Inside a cylindrical device, 80 female mosquitoes were placed with seven to ten days of life per infection, with glucose deprivation for 24 h before the onset of infection. A methodology using an artificial glass feeder, as proposed for sandflies, was attached to the appropriate opening of the mosquito cage and wrapped in a butcher’s sausage membrane [41].

The feeder was connected by hoses that allowed the circulation of water heated to 37 °C to keep the blood solution at a similar temperature to mammals. The top of the feeder was filled with a mixture of 2 mL of defibrinated sheep’s blood plus 2 mL of cell suspension infected with CHIKV. After feeding, engorged females were placed in individual cages and fed bee honey diluted in 10% water, and those that did not feed during the experiment were discarded from the study. Female mosquitoes that had previously fed on infected blood were removed from the cage 14 days post-infection (14 DPI) and stored in a −70 °C freezer in 15 mL falcon tubes.

Samples were grouped on an entomological table refrigerated at approximately −36 °C into 6 pools in triplicates (18 final pools), containing 1, 5, 10, 20, 30, or 40 mosquitoes per pool. Uninfected females were also separated for the negative control in the same pool organization, but without replicates.

### 4.4. Mosquitoes Maceration

Each pool was added to 1 mL of Dulbecco’s phosphate-buffered saline (DPBS) (Life Technologies, Carlsbad, CA, USA) solution containing 0.75% bovine albumin, penicillin (100 IU/mL), and streptomycin (100 µg/mL). A 3 mm tungsten bead was added to each sample, which were macerated using a TissueLyser II (Qiagen, Hilden, Germany) for 1 min at 25 Hz. The samples were then centrifuged at 10,000 rpm for 10 min at 4 °C [42].

### 4.5. RNA Extraction

In order to compare the effectiveness and quality of RNA extraction of CHIKV-infected mosquitoes, four different kits were tested: QIAamp*^®^* Viral RNA Mini Kit (Qiagen); Maxwell*^®^* 16 Viral Total Nucleic Acid Purification kit (Promega, Madison, WI, USA); PureLink*^®^* RNA Mini Kit (Thermo Fisher Scientific, Waltham, MA, USA) with TRIzol*^®^* (Thermo Fisher Scientific); and the PureLink*^®^* RNA Mini Kit (Thermo Fisher Scientific). The four methods were carried out according to the manufacturers’ descriptions. After centrifugation, the supernatant was removed and aliquoted in exact volumes for each method: 140 μL, 200 μL, 300 μL, and 300 μL, respectively, and totalizing 72 aliquots. These four kits are commonly used in the daily routine of the Molecular Biology Laboratory of SAARB/IEC.

### 4.6. RT-qPCR

The reaction was performed using the SuperScript^®^III Platinum^®^One-Step RT-qPCR kit (Thermo Fisher Scientifc) and a set of primers and probes specific to the CHIKV NSP1 gene, as described by [43]. The 25 μL reactions were composed of 12.5 μL of a 2X master mix, 5.5 μL of nuclease-free water, 1.0 μL of each primer, 0.5 of probe, 0.5 μL of SuperScriptIII RT/Platinum Taq Enzyme mix, and 5 μL of the RNA. As a non-competitive endogenous control, we used a set of primers and probes specific to the rps17 gene [44].

In a AriaMx Real-time PCR System (Agilent, Santa Clara, CA, USA), the assays were conducted under the following cycling conditions: an initial reverse transcription step at 50 °C for 30 min; a denaturation step at 95 °C for 2 min; 45 cycles of 15 s at 95 °C; and a 1 min final extension step at 60 °C. Samples were analyzed in duplicate and considered positive when the cycle threshold (Ct) value was less than 38. Besides the endogenous control detection, tests were validated using a positive (CHIKV-infected mice brain) and a negative control (nuclease-free water).

### 4.7. RNA Quantification and Purity

The RNA concentration was quantified using two different apparatuses: Qubit 4 Fluorometer (Thermo Fisher Scientifc); and NanoDrop^®^ One/OneC Microvolume UV-Vis Spectrophotometer (Thermo Fisher Scientifc). The latter also provided information about RNA purity.

### 4.8. Statistical Analysis

Each variable normality distribution was assessed using the Shapiro–Wilk test. The variances’ homogeneity was checked using Levene’s test, while the outliers’ detection was conducted by analyzing boxplots and using the quartile and interquartile range (IQR) method.

The Student’s *t*-test for independent samples was applied to check differences between the means. In addition, the Kruskal–Wallis non-parametric analysis of variance test, followed by Dunn’s post hoc test with Bonferroni correction for multiple comparisons, was used to compare the variables’ medians in the different datasets. Spearman’s correlation test was used to assess the correlations between the number of mosquitoes per pool and the Ct mean value. For all statistical analyses, *p*-values < 0.05 and a 95% confidence interval were considered significant.

Data analysis was conducted in R software version 4.3.2 [45] using the dplyr, forcats, reshape, and ggplot2 packages from the R tidyverse library [46] for data processing and graph plotting, respectively. The Student’s *t*-test and Kruskal–Wallis test were carried out using the R base package [45], while Levene’s test was applied using the R rstatix package [47], and Spearman’s correlation analysis used the R library Hmisc [48].

### 4.9. Ethical Approval

This research was approved by the Ethics Committee for the Use of Animals (CEUA) of the IEC on 6 July 2023, under certificate no. 14/2023, since hamster and sheep blood were used, respectively, for the mosquito colonies’ maintenance and infection.

## 5. Conclusions

To ensure accurate RT-qPCR results and increase sensitivity, extraction methods must be extremely efficient, provide high-quality RNA, produce a reasonable amount of RNA, and, in general, be consistent and robust. Our study shows the highly effective nature of two extraction protocols, namely, QIAamp^®^ and PureLink^®^ with TRIzol^®^, over the others, which may provide help in CHIKV entomovirological surveillance planning in endemic and epidemic areas and others with a higher risk of CHIKV importation.

## Figures and Tables

**Table 1:** RT-qPCR results via RNA extraction kit.

Pool	QIAamp^®^	Maxwell^®^	PureLink^®^ with TRIzol^®^	PureLink^®^
1A	+	+	+	+
1B	+	+	+	-
1C	+	-	+	+
5A	+	-	+	+
5B	+	-	+	+
5C	+	-	+	+
10A	+	+	+	+
10B	+	-	+	+
10C	+	-	+	+
20A	+	-	+	+
20B	+	-	+	+
20C	+	+	+	+
30A	+	-	+	-
30B	+	+	+	-
30C	+	+	+	+
40A	+	+	+	-
40B	+	+	+	+
40C	+	-	+	-

+: detected; -: undetected.

**Table 2:** Spearman’s correlation results.

Pair of Variables	Correlation Coefficient (ρ)	*p*-Value	Correlation Level	Statistical Significance
n and QIAamp^®^	−0.7492752	0.0003448	strong	***
n and Maxwell^®^	−0.003441734	0.9892	very weak	non-significant
n and PureLink^®^ with TRIzol^®^	−0.5423624	0.02005	moderate	*
n and PureLink^®^	−0.2566004	0.304	weak	non-significant

n: number of mosquitoes per pool; *: *p* < 0.05 (significant); ***: *p* < 0.0005 (highly significant).

**Table 3:** Comparative table of tested kits.

Factor	QIAamp^®^	Maxwell^®^	PureLink^®^ with TRIzol^®^	PureLink^®^
Cost per sample	USD 7.30	USD 14.90	USD 8.75	USD 7.04
Efficiency	Very efficient	Not very efficient	Efficient	Not very efficient
Sensitivity	High	Very Low	High	Low
Time per sample	20 min	65 min	28 min	8 min
Type of extraction	Manual	Manual and automated	Manual	Manual

## Data Availability

Data is contained within the article and Supplementary Materials.

## Supplementary Materials

The following supporting information can be downloaded at https://www.mdpi.com/article/10.3390/ijms25126700/s1.
